# Inertial moment and axis misalignment are relevant sources of error in isokinetic dynamometry

**DOI:** 10.1007/s00421-026-06194-w

**Published:** 2026-04-29

**Authors:** Zhenya Smirnov, Dennis Mehley, Falk Mersmann, Adamantios Arampatzis, Sebastian Bohm

**Affiliations:** 1 Department of Training and Movement Sciences, Humboldt-Universität zu Berlin, Berlin, Germany; 2 Berlin School of Movement Science, Humboldt-Universität zu Berlin, Berlin, Germany

**Keywords:** Plantarflexion contractions, Dynamometry, Moment correction, Inertial moment, Axis misalignment

## Abstract

Human in vivo determination of the moment-angle-angular velocity and muscle force-length-velocity relationships is typically based on dynamometry. Although inertial moment and axis misalignment are inevitable during active joint rotations, their effect on measurement errors during dynamic plantarflexions is unknown. We studied the effect of moment corrections during concentric plantarflexions at five pre-set angular velocities ranging from 45^o^/s to 270^o^/s and at the maximal and two submaximal levels of soleus muscle activation in fourteen healthy young participants. Omitting moment corrections led to considerable errors in the maximum measured moment of up to 39.5% and 86.5% in maximal and submaximal plantarflexions, respectively. Measurement errors also affected the time instants and ankle joint angles at which moment maxima were identified. The contribution of inertial moment and axis misalignment corrections to the occurring errors had a large inter-individual variability and depended both on angular velocity and muscle activation level. The contribution of inertial moment increased with angular velocity and muscle activation level, while the contribution of axis misalignment decreased with angular velocity and increased with muscle activation level. Therefore, we posit a strong necessity to account for these two often neglected corrections during dynamic plantarflexions.

## Introduction

Force-length and force-velocity relationships are considered basic force generation properties of skeletal muscle. To derive these relationships in vivo, joint moment is required to be determined, which is usually done by dynamometry. Previous research pointed out that the moment measured by a dynamometer needs to be corrected for the gravitational and inertial properties of the dynamometer adapter and actuated body segments, the misalignment between the dynamometer and joint axes of rotation and passive joint moment (Winter et al. 1981; Kaufman et al. 1995; Arampatzis et al. 2005; Yoon and Mansour 1982). While correcting for gravitational and passive joint moment has become a standard procedure, inertial moment and axis misalignment corrections are usually still omitted.

One reason why the correction of inertial moment is bypassed in dynamic measurements is the unknown inertial properties of the dynamometer adapter. This issue can be circumvented by modelling (Bobbert and Harlaar 1993; Baltzopoulos 1995). However, the modelling approach involves simplifying assumptions about the geometric characteristics and mass distribution of the adapter, and its reliability is uncertain. Another reason why inertial moment correction is assumed to be unnecessary in apparently isokinetic measurements is that inertial moment is zero at a constant angular velocity. Simultaneously, maximum moments (Martin et al. 1994; Ferri et al. 2003; Paulauskas et al. 2020) and moments at certain joint angles (Thom et al. 2007; Baxter and Piazza 2014; Holzer et al. 2023) are commonly used for assessing joint moment generation capacity, whereas intervals of constant angular velocity may not be achieved during measurements at high pre-set angular velocities or may not include moment maxima (Iossifidou and Baltzopoulos 1998; Csapo et al. 2010) or the joint angles of interest. Such methodological drawbacks and inconsistencies may introduce significant errors into measurement data (Iossifidou and Baltzopoulos 1998) and decrease the validity of reported results.

The issue of the misalignment between the dynamometer and joint axes of rotation has also been highlighted previously (Arampatzis et al. 2004, 2005, 2007; Tsaopoulos et al. 2011). It has been reported that unavoidable displacements between the dynamometer and joint axes of rotation during maximal contractions may lead to substantial differences in the lever arms of adapter reaction force about the two axes, which may result in considerable errors in joint moment assessment (Arampatzis et al. 2004, 2005, 2007; Tsaopoulos et al. 2011). It has also been reported that the effect of axis misalignment on measured moment might depend on the contraction type, joint angle studied and muscle activation level (Arampatzis et al. 2007; Tsaopoulos et al. 2011). Summarizing, it could be argued that the inertial properties of the dynamometer adapter and the misalignment between the dynamometer and joint axes of rotation contribute to errors in the assessment of joint moment and this contribution may depend on angular velocity and muscle activation level. The information on how angular velocity and muscle activation affect measured moment during dynamic plantarflexions is, however, missing.

Plantarflexor muscles play a distinct role in movement performance, economy and stability (Hamner and Delp 2013; Arampatzis et al. 2006; Bohm et al. 2021; Dick et al. 2019). Their moment/force production properties are often assessed employing dynamic contractions with inertial moment and axis misalignment corrections being bypassed (Thom et al. 2007; Monte et al. 2020). This study aimed to evaluate the influence of angular velocity and muscle activation level on the contribution of dynamometer adapter and foot gravitational and inertial moments, axis misalignment and passive ankle joint moment to the moment measured by the dynamometer during dynamic plantarflexions. A secondary objective was to implement an experimental approach to determine the moment of inertia of the dynamometer adapter. We hypothesized that the contribution of inertial moment correction would increase with increasing angular velocity due to longer acceleration and deceleration phases and that the contribution of axis misalignment correction would decrease with increasing angular velocity and decreasing muscle activation due to a decreased moment applied by the foot to the dynamometer adapter.

## Methods

### Participants and experimental protocol

Fourteen physically active young adults were recruited for the study (5 females/9 males, age: 26.7 ± 3.7 years, body mass: 69.6 ± 13.5 kg, body height: 175.9 ± 9.8 cm). None of the participants reported any injuries of the right (investigated) leg 12 months prior to the measurements or any neuromuscular impairments. After being informed about the purposes and measurement procedures of the study, participants gave written informed consent according to the Declaration of Helsinki. The study was approved by the ethics committee of the Humboldt-Universität zu Berlin (HU-KSBF-EK_2024_0006). Participants accomplished two training sessions and one following data collection session within 11 days with at least one day off between sessions. In the first training session, participants first got accustomed to maximal and submaximal fixed-end plantarflexions (Bohm et al. 2024) and then practiced maximal dynamic plantarflexions and submaximal dynamic plantarflexions at 60 to 70% of the maximum electromyographic activity of the soleus muscle (EMG_max_) at five pre-set angular velocities (45, 90, 150, 210 and 270°/s). In the second training session, participants practiced submaximal dynamic plantarflexions at 30 to 40% of EMG_max_ at all five angular velocities (how EMG_max_was established is described below).

Participants were placed into a prone position on the isokinetic dynamometer (System 3, Biodex Medical Inc., Shirley, NY, USA) with their right knee flexed to approximately 110^o^ (0^o^ refers to the knee fully extended; Bohm et al. 2024). This knee angle was chosen to exclude the contribution of the gastrocnemius medialis et lateralis muscle to the generated ankle joint moment during plantarflexions (Hof and van den Berg 1977; Rubenson et al. 2012). We defined a participant’s neutral ankle joint angle (90^o^) as the angle at which the sole of their foot was orthogonal to the midline of the shank (ankle angles less than 90^o^ refer to plantarflexion and ankle angles more than 90° refer to dorsiflexion). The maximum angle at which soleus EMG activity remained at the level of the inactive state during a passive dorsiflexion, as determined by visual inspection, was set as the maximum dorsiflexion angle for a particular participant. Each participant’s range of ankle rotation was defined as the range between this maximal dorsiflexion angle and 70° (plantarflexion). In each session, participants performed a standardized warm-up consisting of several fixed-end plantarflexions: ten at 40% and six at 70% of their self-perceived maximum effort in a consecutive manner, and three maximal plantarflexions with at least 30-second rest in between. These contractions were executed at a fixed ankle angle between 90° to 110°.

The experimental procedure consisted of series of maximal and submaximal plantarflexions at five randomized angular velocities of 45, 90, 150, 210 and 270°/s without preload. For each angular velocity, participants started with a series of three maximal plantarflexions. After that, an angular velocity-specific EMG_max_ was established because joint angular velocity may affect the EMG activity (Westing et al. 1991; Bobbert and Harlaar 1993). Participants were then asked to perform six series of 10 to 12 submaximal dynamic plantarflexions: three series at 60 to 70% of EMG_max_ and then three series at 30 to 40% of EMG_max_. The ongoing EMG signal was displayed as a percentage of EMG_max_ on a monitor to guide participants for the target EMG levels (Bohm et al. 2024). The displayed EMG signal was processed first by applying a second-order high-pass Butterworth filter with a cut-off frequency of 20 Hz, then rectifying and averaging over 50 ms. During the trials, each participant’s foot was fastened to the adapter footplate around the metatarsophalangeal joints in order for the participant to be able to dorsiflex by themselves. A rest period of at least two minutes was allowed between series. The measurements were performed at the hardest end stop cushion setting of the dynamometer (1 out of 9).

## Ankle joint moments

Moment was measured by the dynamometer at 1000 Hz and filtered with a second-order low-pass Butterworth filter with a cut-off frequency of 6 Hz. Measured moment ${M}_{\mathrm{meas}}$ was then corrected for (a) the gravitational and inertial effects of the dynamometer adapter, (b) the misalignment between the dynamometer and ankle joint axes of rotation, (c) the gravitational and inertial effects of the foot and the passive moment of the ankle joint. Kinematic data was acquired via a motion capture system (Vicon Nexus, version 2.15, Vicon Motion Systems, Oxford, UK) integrating eleven cameras operating at 250 Hz. Six reflective markers (14 mm diameter) were placed onto the second metatarsophalangeal joint, the medial and lateral malleoli, the medial and lateral femoral epicondyles and a point at the straight line connecting the lateral femoral epicondyle and the greater trochanter of the right leg. One additional reflective marker (6 mm diameter) was placed over the Achilles tendon insertion to the calcaneus. Five further reflective markers (14 mm diameter) were placed onto the dynamometer adapter to capture its motion: three markers onto the footplate, one onto the rotation axis and one onto the lever. Kinematic data was first filtered with a second-order low-pass Butterworth filter with a cut-off frequency of 6 Hz and then spline-interpolated to 1000 Hz.

**Fig. 1:**
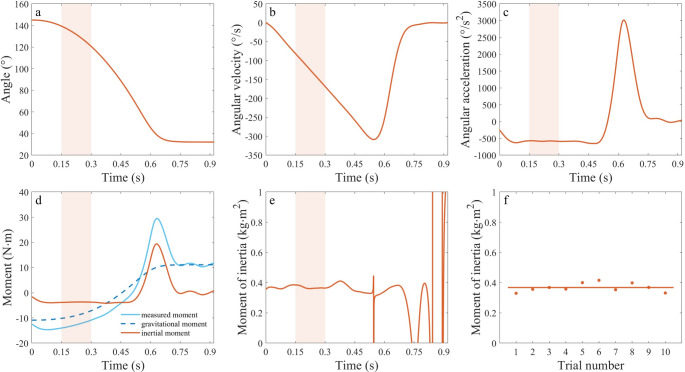
Determination of the adapter moment of inertia. The adapter was rotated ten times at the pre-set angular velocity of 300°/s over a range of motion exceeding that of a participant. Adapter rotation angle (**a**) from each of ten trials was differentiated to derive its angular velocity (**b**) and angular acceleration (**c**). Adapter gravitational moment was subtracted from measured moment to determine adapter inertial moment (**d**). Inertial moment was divided by angular acceleration (**e**), and the result was averaged over the time interval 0.15 to 0.3 s after the rotation onset (shaded area, **a**–**e**) to compute the adapter moment of inertia. The values of the moments of inertia from ten trials were averaged, and the mean value represented the adapter moment of inertia (**f**)

To assess the gravitational contribution of the adapter, moment was measured when the foot adapter was passively rotated at the angular velocity of 5°/s over an angle range exceeding that at which participants performed plantarflexions. To calculate the moment of inertia of the adapter, it was rotated at the angular velocity of 300°/s. The values of the moment of inertia were calculated by dividing inertial moment (i.e. the difference between the moment measured during a passive rotation at 300°/s and the gravitational moment of the adapter at the same angle) by the angular acceleration of the adapter at the plateau of angular acceleration (between 150 and 300 ms after the rotation onset) and by averaging these values over the plateau (Fig. 1). We performed ten rotations at 300°/s, and the average value of the moments of inertia over ten trials represented the adapter moment of inertia ${I}_{\mathrm{dyn}}$. For active plantarflexion trials, the inertial component ${M}_{\mathrm{inert}}$ of measured moment was calculated as the product of ${I}_{\mathrm{dyn}}$ and adapter angular acceleration ${\dot{\omega}}_{\mathrm{dyn}}$. Then, this value as well as adapter gravitational moment ${M}_{\mathrm{gravi}}$ was subtracted (with an appropriate sign) from measured moment ${M}_{\mathrm{meas}}$:

$${M}_{\mathrm{leg}}={M}_{\mathrm{meas}}-{M}_{\mathrm{gravi}}-{M}_{\mathrm{inert}}={M}_{\mathrm{meas}}-{M}_{\mathrm{gravi}}-{I}_{\mathrm{dyn}}\cdot{\dot{\omega}}_{\mathrm{dyn}}.$$

Further, the misalignment between the dynamometer axis of rotation and the ankle joint axis of rotation was corrected according to an established procedure to determine ankle joint resultant moment (Arampatzis et al. 2005):

$${M}_{\mathrm{ankle}}=\frac{{l}_{\mathrm{a}}}{{l}_{d}}{M}_{\mathrm{leg}},$$

where ${l}_{\mathrm{d}}$ and ${l}_{\mathrm{a}}$ are the lever arms of the force applied by the foot to the adapter about the dynamometer axis of rotation and about the ankle joint axis of rotation, respectively. The point of reaction force application was defined as the projection of the second metatarsophalangeal joint marker onto the surface of the footplate in the plane orthogonal to the dynamometer axis (sagittal plane). The surface of the footplate was reconstructed from the footplate markers and their known radius.

Last, we created a model of each participant’s foot by estimating its mass, moment of inertia and center-of-mass position according to Dempster (1955). Based on this data, we approximated foot gravitational and inertial moments about the ankle joint axis of rotation. To estimate the passive moment ${M}_{\mathrm{pass}}$ exerted by the ankle joint during plantarflexions, each participant’s ankle joint was passively rotated at the angular velocity of 5°/s (Arampatzis et al. 2005) and measured moment underwent the corrections just described. To compute active ankle (corrected) joint moment ${M}_{\mathrm{corr}}$, passive joint moment and the sum ${M}_{\mathrm{foot}}$ of foot gravitational and inertial moments were subtracted (accounting for the sign) from ${M}_{\mathrm{ankle}}$ (Arampatzis et al. 2007):

$${M}_{\mathrm{corr}}={M}_{\mathrm{ankle}}-{M}_{\mathrm{pass}}-{M}_{\mathrm{foot}}.$$

## Electromyographic activity and muscle activation dynamics

Two surface EMG sensors (Myon 320, Myon, Schwarzenberg, Switzerland; electrodes Kendall H124SG, Cardinal Health, Waukegan, IL, USA) were placed on the soleus and tibialis anterior muscles with an inter-electrode center-to-center distance of 24 mm. EMG activity was acquired at 1000 Hz. The raw EMG signals were processed first by applying a second-order high-pass Butterworth filter with a cut-off frequency of 20 Hz, then rectifying and applying a second-order low-pass Butterworth filter with a cut-off frequency of 6 Hz. Prior to determining the angular velocity-specific maximum EMG activity during maximal dynamic plantarflexions, a moving average over 50 ms was applied to the processed EMG signals.

To model muscle activation dynamics $\hat{a}\left(t\right)$, processed EMG signals were first normalized to the angular velocity-specific EMG maximum. Then, the normalized signals served as the inputs $u\left(t\right)$ to the first-order linear ordinary differential equation proposed by Zajac (1989):

$$\frac{d\hat{a}\left(t\right)}{dt}=-\frac{1}{{\tau}_{\mathrm{act}}}\left(\beta+\left(1-\beta\right)u\left(t\right)\right)\hat{a}\left(t\right)+\frac{1}{{\tau}_{\mathrm{act}}}u\left(t\right).$$

Similarly to Dick et al. (2017), the activation constant ${\tau}_{\mathrm{act}}$ was calculated as a linear combination of activation constants specific to fast and slow muscle fibers (${\tau}_{\mathrm{act,f}}=25$ ms and ${\tau}_{\mathrm{act,s}}=45$ ms):

$${\tau}_{\mathrm{act}}={p}_{\mathrm{f}}{\tau}_{\mathrm{act,f}}+{p}_{\mathrm{s}}{\tau}_{\mathrm{act,s}}.$$

The proportion of fast (${p}_{\mathrm{f}})$ and slow ${(p}_{\mathrm{s}})$ fibers of the soleus muscle (Johnson et al. 1973; Edgerton et al. 1975) was taken as the coefficients of this linear combination (${p}_{\mathrm{f}}=0.22$ and ${p}_{\mathrm{s}}=0.78$). The activation-deactivation ratio $\beta$ was set to $0.6$ as independent of muscle fiber type (Dick et al. 2017).

### Data analysis

**Fig. 2:**
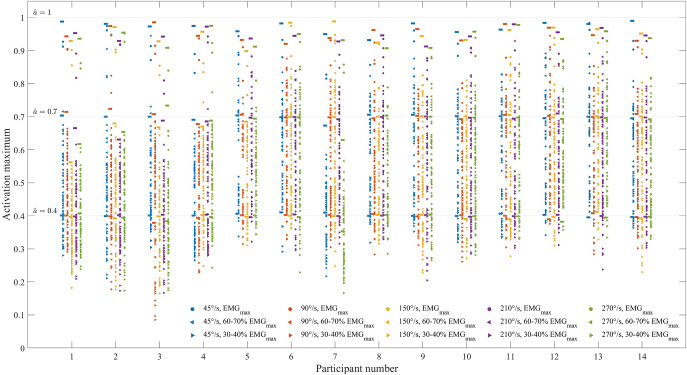
Selection of plantarflexions with the activation maxima closest to the target levels. At each of five pre-set angular velocities, participants first performed three maximal dynamic plantarflexions without preload. After the EMG maximum (EMG_max_) was identified (see the main text), participants performed three series of 10–12 plantarflexions at the EMG levels of 60 to 70% EMG_max_ and three series of 10–12 plantarflexions at the EMG levels of 30 to 40% EMG_max_. The EMG signal normalized to the angular velocity-specific EMG_max_ was used as an input to the first-order linear ordinary differential equation proposed by Zajac (1989). Among the three maximum plantarflexions, the one with the maximum activation (closest to ${\hat{a}}_{\mathrm{max}}=1$) was selected for further analysis. Among the plantarflexions performed at the EMG levels of 60 to 70% EMG_max_, the one with the activation maximum closest to ${\hat{a}}_{\mathrm{0.7}}=0.7$ was taken for further analysis. Finally, among the plantarflexions performed at the EMG levels of 30 to 40% EMG_max_, the one with the activation maximum closest to ${\hat{a}}_{\mathrm{0.4}}=0.4$ was taken for further analysis. The activation maxima reached during all single plantarflexions are shown in the figure. The plantarflexions selected for the analysis are marked with dashes

The data was processed and analyzed in MATLAB (version R2023a, MathWorks, Natick, MA). The analyzed range of motion (onset and termination) of each dynamic plantarflexion was determined from the kinematic data. The onset of each plantarflexion was defined as the maximum angle reached by the participant during the relaxed dorsiflexion preceding the plantarflexion. The time instant at which ankle joint angular velocity was 5°/s during the deceleration phase of the plantarflexion corresponded to its termination. From all plantarflexions performed by each participant at a particular angular velocity, we selected three plantarflexions which activation maxima were closest to the three target activation levels: ${\hat{a}}_{\mathrm{max}}=1$, ${\hat{a}}_{\mathrm{0.7}}=0.7$ and ${\hat{a}}_{\mathrm{0.4}}=0.4$ (one plantarflexion for each activation level; Fig. 2).

Axis misalignment correction moment was defined as the difference of moments before and after axis misalignment correction:

$${M}_{\mathrm{axis}}={M}_{\mathrm{leg}}-{M}_{\mathrm{ankle}}=\left(1-\frac{{l}_{\mathrm{a}}}{{l}_{\mathrm{d}}}\right){M}_{\mathrm{leg}}.$$

All moment corrections ${M}_{\mathrm{gravi}}$, ${M}_{\mathrm{inert}}$, ${M}_{\mathrm{axis}}$, ${M}_{\mathrm{foot}}$ and ${M}_{\mathrm{pass}}$ as well as measured ${M}_{\mathrm{meas}}$ and active ankle joint ${M}_{\mathrm{corr}}$ moments were normalized to the participant’s body mass for further analysis (the same designations are used for body-mass normalized moments further in the text).

We analyzed the absolute ($\left|{M}_{\mathrm{meas,max}}-{M}_{\mathrm{corr,max}}\right|$) and relative ($\left|{M}_{\mathrm{meas,max}}-{M}_{\mathrm{corr,max}}\right|/{M}_{\mathrm{meas,max}}$) differences between measured and corrected moment maxima as well as the distribution of the time instants and ankle joint angles at which ${M}_{\mathrm{meas}}$ and ${M}_{\mathrm{corr}}$ reached their maxima. The Wilcoxon signed-rank tests with the Benjamini-Hochberg correction with a false discovery control level ${q}^{\mathrm{*}}=0.05$ and accounting for $K=15$ combinations of angular velocities and activation levels was used to sequentially ($p_{k} \leqslant k q^{*} /K,k = 1..K$) compare the time instants of ${M}_{\mathrm{meas,max}}$ and ${M}_{\mathrm{corr,max}}$ and the corresponding ankle joint angles for each combination.

We used time-moment curves to assess relative contributions $C\left({M}_{\mathrm{i}}\right)$ of each moment correction ${M}_{\mathrm{i}}$ to the overall correction effect. Since each correction can be considered a projection of the five-dimensional vector ${M}_{\mathrm{effect}}=\left({M}_{\mathrm{gravi}},{M}_{\mathrm{inert}},{M}_{\mathrm{axis}},{M}_{\mathrm{foot}},{M}_{\mathrm{pass}}\right)$ onto the corresponding axis at each time instant, we divided the squared value of each correction to the sum of squared values of all corrections to compute $C\left({M}_{\mathrm{i}}\right)$, representing the contribution of each correction to the length of ${M}_{\mathrm{effect}}$:

$$C\left({M}_{\mathrm{i}}\right)=\frac{{M}_{\mathrm{i}}^{2}}{\sum\limits_{\mathrm{j}=1}^{5}{M}_{\mathrm{j}}^{2}}.$$

We averaged $C\left({M}_{\mathrm{i}}\right)$ values for each plantarflexion of interest over its duration ($\bar{C}\left({M}_{\mathrm{i}}\right)$). After pooling $\bar{C}\left({M}_{\mathrm{i}}\right)$ values for all combinations of angular velocities and activation levels together, we found that the data well followed a gamma distribution (with correction-specific parameters). Then, we applied generalized linear mixed-effects models to $\bar{C}\left({M}_{\mathrm{inert}}\right)$ and $\bar{C}\left({M}_{\mathrm{axis}}\right)$ to account for the effects of angular velocity and activation level (fixed-effect predictors) and participant number (random-effect predictors). A nested natural logarithm and the natural logarithm were used as link functions in the two models, correspondingly (loglog and log functions in MATLAB). Adapter gravitational, foot and passive joint corrections were excluded from this analysis. It can be argued that gravitational moment is independent of angular velocity and muscle activation and passive joint moment is not directly attributed to active muscle force production which is predicated by muscle activation and underlies dominant changes of joint angular velocity. Besides that, the angular velocity-dependent fraction of passive joint moment can be considered small to negligible (Gajdosik 1997; Gajdosik et al. 2005; Johns and Wright 1962). Foot moment correction was excluded from the analysis because of its negligible contribution to the overall correction effect. Additionally, we applied the latter formula to the moment corrections averaged for all participants (${\bar{M}}_{\mathrm{i}}$) to visualize the contribution of single corrections.

## Results

**Fig. 3:**
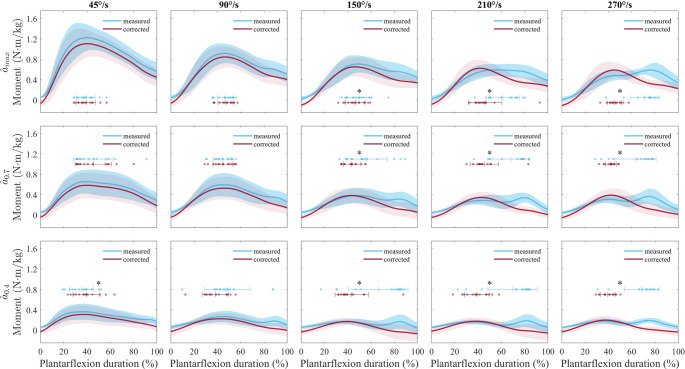
Normalized-to-body mass measured ${M}_{\mathrm{meas}}$ and corrected ${M}_{\mathrm{corr}}$ plantarflexion moments at five angular velocities (45°/s, 90°/s, 150°/s, 210°/s and 270°/s) and three activation levels (${\hat{a}}_{\mathrm{max}}$, ${\hat{a}}_{\mathrm{0.7}}$ and ${\hat{a}}_{\mathrm{0.4}}$). The curves and shaded areas represent mean±standard deviation. Light blue and burgundy dots represent the time instants of individual ${M}_{\mathrm{meas}}$ and ${M}_{\mathrm{corr}}$ maxima, respectively. The horizontal axis is normalized to the duration of plantarflexion. *Statistically significant differences in the time instants of ${M}_{\mathrm{meas}}$ and ${M}_{\mathrm{corr}}$ maxima

Maximal differences between measured and corrected moment maxima at the five angular velocities were 0.27 Nm/kg at ${\hat{a}}_{\mathrm{max}}$, 0.19 Nm/kg at ${\hat{a}}_{\mathrm{0.7}}$ and 0.18 Nm/kg at ${\hat{a}}_{\mathrm{0.4}}$, while corrected moment maxima ranged from 0.34 to 1.45 Nm/kg, from 0.12 to 1.19 Nm/kg and from 0.03 to 0.79 Nm/kg at the three activation levels (Table 1). The largest absolute differences of 0.26 Nm/kg and 0.27 Nm/kg were observed correspondingly at 45°/s and 270°/s at ${\hat{a}}_{\mathrm{max}}$. Relative differences between measured and corrected moment maxima ranged from 0 to 86.5% among all angular velocities and activation levels, being largest at 150°/s and 210°/s at ${\hat{a}}_{0.4}$ (Table 1). Moment corrections also changed the time instants and ankle joint angles at which moment maxima were achieved (Fig. 3; Table 2). The time instants of corrected moment maxima tended to reside in the first half of the plantarflexions, while the time instants of measured moment maxima were more broadly distributed. The shift of the time instants of moment maxima was pronounced at higher angular velocities at all three activation levels (Fig. 3; Table 2). The ankle joint angles at which moment maxima were achieved shifted to more dorsiflexed angles in the same conditions where significant shifts of time instants occurred (Fig. 3) with the addition of the angular velocity of 90°/s at the maximum activation level.

**Table 1:** Absolute and relative differences between the maxima of measured and corrected plantarflexion moments at the five angular velocities (45°/s, 90°/s, 150°/s, 210°/s and 270°/s) and the three activation levels (${\hat{a}}_{\mathrm{max}}$, ${\hat{a}}_{\mathrm{0.7}}$ and ${\hat{a}}_{\mathrm{0.4}}$) (mean±standard deviation)

Activation level		45°/s	90°/s	150°/s	210°/s	270°/s
${\hat{a}}_{\mathrm{max}}$	Absolute differences (Nm/kg) Relative differences (%)	0.14 ± 0.08 [0.01, 0.26] 11 ± 6.5 [0.9,23.3]	0.09 ± 0.06 [0,0.2] 9.6 ± 6.6 [0,22.7]	0.09 ± 0.07 [0,0.2] 11.8 ± 8.9 [0.2,25.6]	0.09 ± 0.06 [0.01,0.17] 13.6 ± 7.9 [0.8,22.6]	0.14 ± 0.05 [0.07,0.27] 22.1 ± 8 [10.6,39.5]
${\hat{a}}_{0.7}$	Absolute differences (Nm/kg) Relative differences (%)	0.08 ± 0.05 [0,0.19] 12.5 ± 8.5 [0.7,28.7]	0.08 ± 0.05 [0.01,0.17] 13.8 ± 9.9 [1.6,34.5]	0.06 ± 0.03 [0.01,0.1] 17.1 ± 12.2 [3.7,46.6]	0.09 ± 0.06 [0.01,0.17] 23.4 ± 15.1 [1.4,47.6]	0.08 ± 0.05 [0,0.17] 23.1 ± 16.5 [0.7,59.3]
${\hat{a}}_{0.4}$	Absolute differences (Nm/kg) Relative differences (%)	0.07 ± 0.04 [0,0.16] 21 ± 14 [0.1,44.7]	0.05 ± 0.04 [0.01,0.1] 21.4 ± 18.2 [1.8,53.9]	0.07 ± 0.06 [0.01,0.18] 30.8 ± 24 [3.6,84.6]	0.06 ± 0.05 [0,0.17] 27.1 ± 22.8 [0.8,86.5]	0.06 ± 0.03 [0.01,0.1] 27.3 ± 14.9 [4.4,50.4]

In brackets are the minimum and maximum differences found between the maxima of measured and corrected plantarflexion moments for all participants (*n* = 14)

**Table 2:** Time instants and ankle joint angles of the maxima of measured and corrected plantarflexion moments at the five angular velocities (45°/s, 90°/s, 150°/s, 210°/s and 270°/s) and the three activation levels (${\hat{a}}_{\mathrm{max}}$, ${\hat{a}}_{\mathrm{0.7}}$ and ${\hat{a}}_{\mathrm{0.4}}$) (mean±standard deviation in percent of the plantarflexion duration, % PF, or in degrees for ankle joint angles)

Activation level		45°/s	90°/s	150°/s	210°/s	270°/s
${\hat{a}}_{\mathrm{max}}$	Time instants of ${M}_{\mathrm{meas,max}}$ (% PF) Time instants of ${M}_{\mathrm{corr,max}}$ (% PF) Joint angles at ${M}_{\mathrm{meas,max}}$ (°) Joint angles at ${M}_{\mathrm{corr,max}}$ (°)	39 ± 8 [29, 57] 39 ± 8 [29, 57] (*p* = 0.091) 88.84 ± 6.99 [78.02, 105.91] 88.76 ± 7.1 [77.87, 105.66] (*p* = 0.110)	47 ± 7 [36, 56] 47 ± 7 [37, 57] (*p* = 0.066) 88.41 ± 5.84 [78.62, 101.43] 88.1 ± 5.93 [78.09, 101.03] (*p* = 0.033)	49 ± 11 [35, 75] 46 ± 8 [32, 59] (*p* = 0.014) 90.43 ± 10.25 [72.5, 111.51] 92.32 ± 8.63 [79.41, 110.94] (*p* = 0.017)	62 ± 13 [37, 80] 46 ± 14 [33, 93] (*p* = 0.002) 82.91 ± 12.17 [68.2, 107.21] 95.31 ± 9.12 [71.76, 111.66] (*p* < 0.001)	71 ± 12 [38, 83] 45 ± 6 [33, 57] (*p* < 0.001) 78.32 ± 12.04 [66.75, 110.05] 98.04 ± 7.52 [88.37, 116.33] (*p* < 0.001)
${\hat{a}}_{0.7}$	Time instants of ${M}_{\mathrm{meas,max}}$ (% PF) Time instants of ${M}_{\mathrm{corr,max}}$ (% PF) Joint angles at ${M}_{\mathrm{meas,max}}$ (°) Joint angles at ${M}_{\mathrm{corr,max}}$ (°)	45 ± 17 [31, 91] 45 ± 16 [31, 80] (*p* = 0.819) 88.55 ± 8.87 [75.55, 107.94] 88.52 ± 8.52 [78.04, 107.84] (*p* = 0.903)	44 ± 8 [30, 56] 45 ± 9 [29, 56] (*p* = 0.340) 90.91 ± 8.62 [79.88, 111.9] 90.73 ± 8.75 [79.53, 111.18] (*p* = 0.305)	56 ± 18 [33, 89] 43 ± 7 [34, 55] (*p* < 0.001) 88.75 ± 12.04 [71.24, 111.07] 96.23 ± 7.03 [85.6, 113.41] (*p* < 0.001)	68 ± 16 [36, 84] 45 ± 12 [30, 83] (*p* < 0.001) 81.33 ± 10.17 [67.21, 98.29] 97.47 ± 10.41 [76.14, 120.18] (*p* < 0.001)	63 ± 18 [29, 79] 42 ± 5 [32, 49] (*p* = 0.005) 84.42 ± 17.11 [67.24, 118.99] 99.2 ± 8.64 [86.39, 116.88] (*p* = 0.005)
${\hat{a}}_{0.4}$	Time instants of ${M}_{\mathrm{meas,max}}$ (% PF) Time instants of ${M}_{\mathrm{corr,max}}$ (% PF) Joint angles at ${M}_{\mathrm{meas,max}}$ (°) Joint angles at ${M}_{\mathrm{corr,max}}$ (°)	35 ± 10 [19, 52] 39 ± 12 [24, 64] (*p* = 0.002) 94.1 ± 7.85 [83.04, 111.36] 92.31 ± 8.01 [77.76, 111.1] (*p* = 0.002)	49 ± 20 [10, 88] 38 ± 11 [13, 56] (*p* = 0.241) 91.37 ± 11.37 [73.06, 122.26] 96.82 ± 10.09 [81.54, 121.56] (*p* = 0.241)	66 ± 25 [17, 89] 43 ± 14 [32, 88] (*p* = 0.009) 82.87 ± 12.92 [68.34, 109.89] 98.92 ± 11.6 [69.77, 118.22] (*p* = 0.007)	70 ± 21 [22, 85] 39 ± 11 [18, 58] (*p* = 0.002) 80.38 ± 12.61 [65.37, 106.36] 100.51 ± 9.54 [83.9, 119.87] (*p* = 0.002)	69 ± 15 [31, 83] 40 ± 6 [29, 50] (*p* < 0.001) 79.32 ± 9.92 [69.39, 102.62] 99.85 ± 8.17 [87.66, 118.01] (*p* < 0.001)

In brackets are the ranges of the time instants and ankle joint angles of the individual maxima of measured and corrected plantarflexion moments for all participants (*n* = 14). Ankle angles more than 90° refer to dorsiflexion and ankle angles less than 90° refer to plantarflexion

**Fig. 4:**
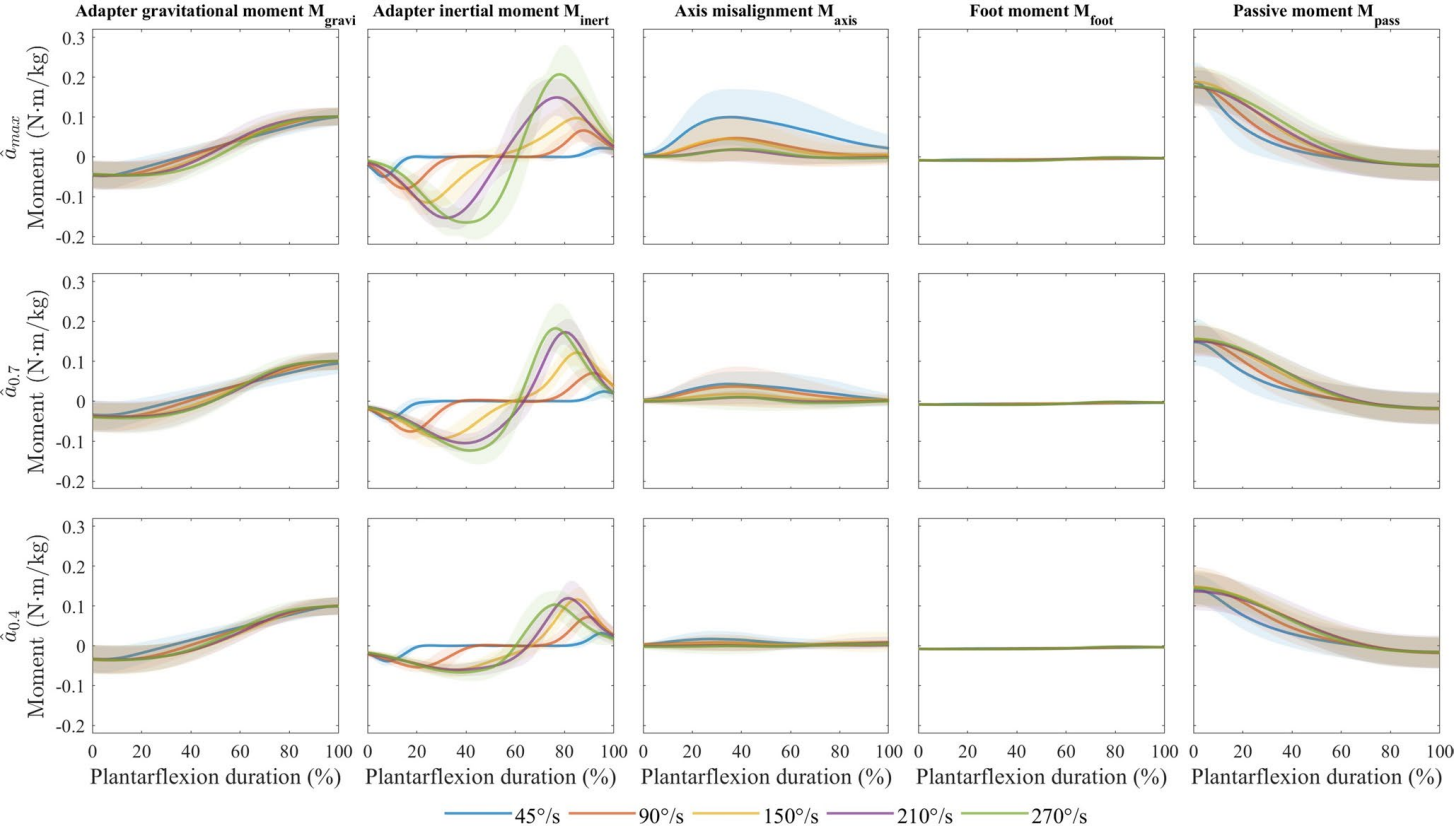
Gravitational adapter moment ${M}_{\mathrm{gravi}}$, adapter inertial moment ${M}_{\mathrm{inert}}$, axis misalignment correction moment ${M}_{\mathrm{axis}}$, the sum of foot gravitational and inertial moments ${M}_{\mathrm{foot}}$ and ankle joint passive moment ${M}_{\mathrm{pass}}$ at the five angular velocities (45°/s, 90°/s, 150°/s, 210°/s and 270°/s) and three activation levels (${\hat{a}}_{\mathrm{max}}$, ${\hat{a}}_{\mathrm{0.7}}$ and ${\hat{a}}_{\mathrm{0.4}}$) averaged over all participants (*n* = 14). The curves and shaded areas represent mean±standard deviation. The horizontal axis is normalized to the duration of plantarflexion

**Fig. 5:**
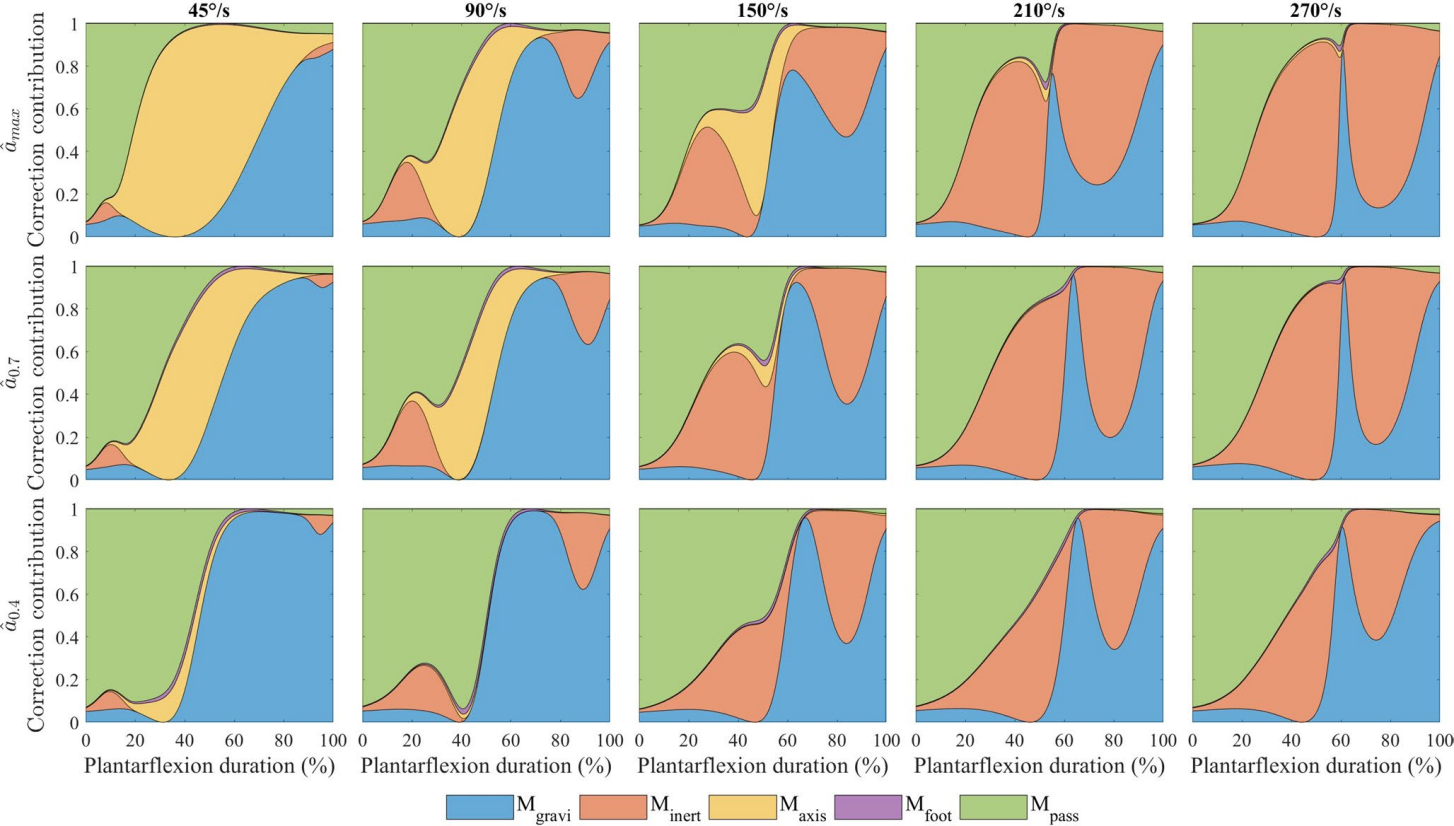
Contributions $C\left(\bar{M}_{\mathrm{i}}\right)$ of average (*n* = 14) gravitational adapter moment ${\bar{M}}_{\mathrm{gravi}}$, adapter inertial moment ${\bar{M}}_{\mathrm{inert}}$, axis misalignment correction moment ${\bar{M}}_{\mathrm{axis}}$, the sum of foot gravitational and inertial moments ${\bar{M}}_{\mathrm{foot}}$ and ankle joint passive moment ${\bar{M}}_{\mathrm{pass}}$ at the five angular velocities (45°/s, 90°/s, 150°/s, 210°/s and 270°/s) and three activation levels (${\hat{a}}_{\mathrm{max}}$, ${\hat{a}}_{\mathrm{0.7}}$ and ${\hat{a}}_{\mathrm{0.4}}$). The horizontal axis is normalized to the duration of plantarflexion

**Fig. 6:**
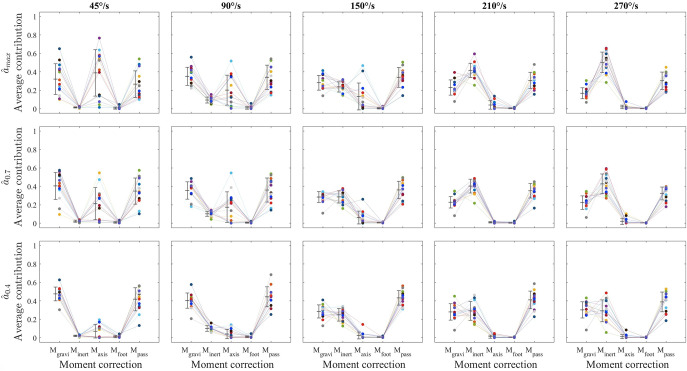
Individual (averaged over the plantarflexion duration) contributions $\bar{C}\left({M}_{\mathrm{i}}\right)$ of gravitational adapter moment ${M}_{\mathrm{gravi}}$, adapter inertial moment ${M}_{\mathrm{inert}}$, axis misalignment correction moment ${M}_{\mathrm{axis}}$, the sum of foot gravitational and inertial moments ${M}_{\mathrm{foot}}$ and ankle joint passive moment ${M}_{\mathrm{pass}}$ at the five angular velocities (45^o^/s, 90^o^/s, 150^o^/s, 210^o^/s and 270^o^/s) and three activation levels (${\hat{a}}_{\mathrm{max}}$, ${\hat{a}}_{\mathrm{0.7}}$ and ${\hat{a}}_{\mathrm{0.4}}$). Bars on the left of the individual values for each moment correction represent group mean±standard deviation. Different colors represent different participants

Angular velocity and muscle activation level influence the average peak-to-peak differences of adapter inertial moment correction and the maxima of axis misalignment correction as well as the maximal and average contributions of these corrections to the overall correction effect. The average peak-to-peak differences of adapter inertial moment correction and the maxima of axis misalignment correction were most affected by angular velocity at ${\hat{a}}_{\mathrm{max}}$ and changed from 0.09 to 0.43 Nm/kg and from 0.12 to 0.04 Nm/kg, correspondingly (Fig. 4). Accordingly, the maximal contribution of the average adapter inertial moment and axis misalignment corrections changed with increasing angular velocity respectively from 0.08 to 0.91 and 0.96 to 0.03 at ${\hat{a}}_{\mathrm{max}}$ (Fig. 5). There was a significant effect of both angular velocity (*p* < 0.001) and muscle activation level (*p* = 0.001) on the average contribution $\bar{C}\left({M}_{\mathrm{inert}}\right)$ of inertial moment correction, as well as significant angular velocity-by-muscle activation level interaction (*p* < 0.001). The contribution of inertial moment correction increased with increasing angular velocity and activation level (Fig. 6). There was a significant effect of both angular velocity (*p* < 0.001) and muscle activation level (*p* < 0.001) on the average contribution $\bar{C}\left({M}_{\mathrm{axis}}\right)$ of axis misalignment correction, as well as significant angular velocity-by-muscle activation level interaction (*p* = 0.023). The contribution of axis misalignment correction decreased with increasing angular velocity and decreasing activation level (Fig. 6).

## Discussion

In the current study, we investigated how angular velocity and muscle activation level influenced the contribution of dynamometer adapter and foot gravitational and inertial moments, axis misalignment and passive ankle joint moment to measured ankle joint moment during dynamic plantarflexions. We also quantified errors that may arise from neglecting corresponding moment corrections. We observed differences between measured and corrected moment maxima reaching 40% during maximal plantarflexions and 87% during submaximal plantarflexions. As hypothesized, the contribution of inertial moment correction increased with angular velocity while the contribution of axis misalignment correction decreased with increasing angular velocity and decreasing muscle activation. Moreover, we observed a decrease in the contribution of inertial moment correction with decreasing muscle activation. Additionally, we developed an experimental approach to determine the moment of inertia of the dynamometer adapter, which is an important parameter for assessing active ankle joint moment during dynamic plantarflexion contractions.

Our observations have important implications for the assessment of dynamic plantarflexions used particularly to estimate the effects of athletic training (Martin et al. 1994; Ferri et al. 2003), therapeutic interventions (Paulauskas et al. 2020) and age-related changes in neuromuscular performance (Thelen et al. 1996; Gajdosik et al. 1999; Dalton et al. 2014). These studies report 13 to 40% improvements or reductions in plantarflexion moment after the intervention or due to aging in most conditions tested. A greater increase of 57% in plantarflexion moment after a resistance training program was reported by Ferri et al. (2003) at the angular velocity of 120°/s, what markedly deviated from 16 to 23% improvements in other conditions in the same study. The range of measured moment errors at the maximum activation level found in the current study ranged from 0 to 40% being 10 to 22% on average. These values are comparable to, or even exceed, the reported effects in some of the aforementioned investigations. Given the contribution of axis misalignment and adapter inertial moment corrections at low and high angular velocities as well as their complex interaction at intermediate velocities, it can be argued that omitting the two corrections could have a noticeable impact on the findings regarding the effects of training and ageing.

Previous studies investigating maximal contractions have already highlighted the necessity to account for inertial moment in dynamic knee extensions (Kaufman et al. 1995; Iossifidou and Baltzopoulos 1998) and for axis misalignment in maximal fixed-end plantarflexions and knee extensions (Arampatzis et al. 2004, 2005; Tsaopoulos et al. 2011) as well as in dynamic plantarflexions at low angular velocities (Arampatzis et al. 2007). Our study corroborates previous observations and complements them by investigating the contribution and interaction of moment corrections during plantarflexions at various angular velocities and muscle activation levels. Kaufman et al. (1995) reported average measurement errors of 20 and 28% (10 and 13% after gravitational correction) at the angular velocities of 60 and 180°/s during maximum knee extensions with inertial moment playing a negligible role at the lower angular velocity and being the main source of error at the higher angular velocity. Iossifidou and Baltzopoulos (1998) reported maximum moments being reached during the deceleration phase during fast knee extensions and attributed their overestimation to inertial moment. We observed similar effects in dynamic plantarflexions with average measurement errors ranging from 10 to 22% at the maximum activation level and with the significant shift of the time instants of moment maxima elicited by the inertial moment of the dynamometer adapter. A tendency towards a larger influence of axis misalignment has been previously observed in fixed-end plantarflexions compared to knee extensions (Arampatzis et al. 2004, 2005; Tsaopoulos et al. 2011). Axis misalignment in maximum fixed-end plantarflexions resulted in the overestimation of measured moment up to 23% (9% on average) compared to 17% overestimation (7% on average) in knee extensions (Arampatzis et al. 2004, 2005). Tsaopoulos et al. (2011) found a 4% overestimation of measured moment during maximum fixed-end knee extensions at the 90° knee angle, but neither at 20° nor during submaximal contractions. The latter is also in line with our results showing a decrease in axis misalignment correction in submaximal plantarflexions.

**Fig. 7:**
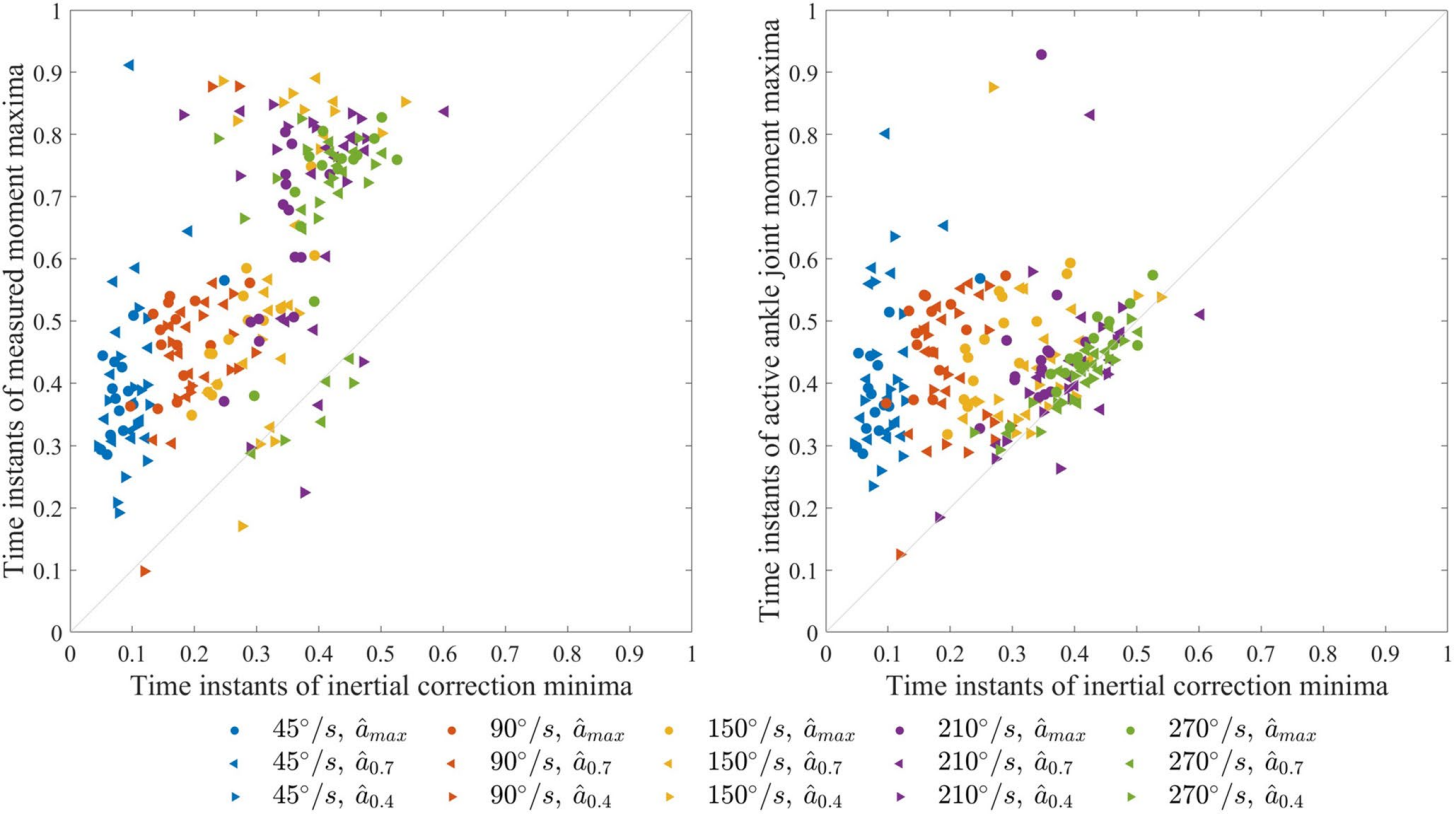
Time instants of measured and active ankle joint maxima in relation to time instants of the minima of adapter inertial moment correction for the five angular velocities (45^o^/s, 90^o^/s, 150^o^/s, 210^o^/s and 270^o^/s), three activation levels (${\hat{a}}_{\mathrm{max}}$, ${\hat{a}}_{\mathrm{0.7}}$ and ${\hat{a}}_{\mathrm{0.4}}$) and all participants (*n* = 14)

Our results indicate that inertial moment and axis misalignment corrections should not be omitted when studying dynamic plantarflexions. Studies employing dynamic plantarflexions usually estimate muscle performance based on the maximum isokinetic moment (Bobbert and van Ingen Schenau 1990; Csapo et al. 2010; Monte et al. 2020) or the moment at the neutral ankle position (Thom et al. 2007; Baxter and Piazza 2014; Holzer et al. 2023). First, it is worth acknowledging that constant angular velocity is not always reached and the maximum measured moment may not coincide with the maximum active ankle joint moment. The prominent shift of the moment maxima at high angular velocities observed in the current study can be attributed to the long acceleration and deceleration phases of the dynamometer adapter. This means, if actual moment maxima and their time instant are aimed to get derived at high angular velocities, inertial moment correction becomes inevitable. Particularly, the time instants of active ankle joint moment maxima at high angular velocities are well aligned with the minima of inertial moment correction while those of measured moment maxima are not (Fig. 7). Second, estimating moment maxima at the neutral ankle position implies that constant angular velocity is reached in that position (Chino et al. 2008). However, whether this condition is fulfilled is inconsistently reported (Thom et al. 2007; Baxter and Piazza 2014; Holzer et al. 2023). Moreover, when estimating moment and force at the maximum muscle shortening velocity is of interest, correcting inertial moment is necessary owing to the fact that fascicle shortening velocity may reach its maximum during the acceleration phase of plantarflexion (Hauraix et al. 2015).

To simplify the estimation of the inertial moment of the dynamometer adapter, we proposed a straightforward and easy-to-implement method to assess the moment of inertia of the adapter not requiring any assumptions about its physical properties and accounting for participant-specific adjustments without any additional calculations. At low angular velocities, axis misalignment may become the main source of error. Despite careful visual alignment and foot fixation, the misalignment between the dynamometer and ankle joint axes of rotation is impossible to prevent over the full course of contraction due to the compliance of the foot/leg soft tissue and the dynamometer (Arampatzis et al. 2005, 2007). A greater joint rotation elicits a larger decrease in the lever arm of the reaction force of the footplate around the ankle joint axis that may lead to substantial differences in moments of this force around the two axes and inconsistencies in moment assessment given a high inter-individual and inter-trial variability of axis misalignment (Arampatzis et al. 2005, 2007). As shown in this study, adapter inertial moment and axis misalignment corrections may also have a complex interaction at intermediate angular velocities and submaximal activation levels, potentially reducing data reliability if omitted. Accounting for angular velocity-dependent and muscle activation-dependent changes in inertial moment and axis misalignment is particularly required when relating ankle joint moment during submaximal plantarflexions to that during maximal ones or when studying fatigue-related changes in plantarflexor moment. Therefore, the inertial moment and axis misalignment corrections should be performed concurrently, especially when aiming for a precise determination of the joint moment-angular velocity or muscle force-velocity relationships, particularly considering the sensitivity of fitting procedures (Edman et al. 1976; Thomas et al. 1987).

Some notes should be made regarding the experimental setup and the limitations of the study. First, our participants accommodated a prone position with the knee flexed compared to a sitting position with the knee extended typically used in plantarflexor strength assessment tests. Bringing the knee in a deep-flexed position is known to influence plantarflexion moment production (Hof and van den Berg 1977; Rubenson et al. 2012; Maganaris 2003); it may also be assumed to attenuate the maximum ankle joint angular velocity due to differences in gastrocnemius and soleus force-velocity relationships (Petrofsky and Phillips 1980), however, the opposite has also been previously reported (Carpentier et al. 1996, 1999). Similarly to previous observations for plantarflexions against considerable inertial loads with the extended knee (Csapo et al. 2010), it was challenging for our participants to reach the pre-set angular velocities of 210°/s and 270°/s. Individual maximum joint angular velocities ranged from 157 to 203°/s (185 ± 11°/s) and from 165 to 240°/s (213 ± 22°/s) at the two pre-set angular velocities at the maximum activation level being significantly different from each other. Maximum joint angular velocities during submaximal plantarflexions were lower than during maximal plantarflexions and had a considerable variability. Despite the differences in joint moment production in the knee-flexed and knee-extended positions, the approach by making gastrocnemius actively insufficient (Hof and van den Berg 1977; Rubenson et al. 2012; Maganaris 2003) allowed us to relate ankle joint moments to soleus activation as precisely as possible, and we expect that our conclusions regarding adapter inertial moment and axis misalignment being relevant sources of measurement error would hold irrespective of the knee joint position. Second, the introduced method for the estimation of the adapter moment of inertia might require a relatively large number of trials. Even though inertial moment several times exceeded the measurement error of the dynamometer, it was relatively small even during fast passive rotations due to a relatively slow angular acceleration. This makes the estimation of the moment of inertia from a single trial or few trials prone to errors. To overcome this issue, we computed the average value of the moment of inertia over ten trials. The coefficient of variation of the moment of inertia value ranged from 3.7 to 11.4% among participants (7.2% on average). Third, the quantification of axis misalignment correction assumed that the reaction force of the footplate was orthogonal to its surface. The non-orthogonality of reaction force could have led to an overestimation of the axis misalignment correction and its contribution to the correction effect at the time instants at which this correction was nondominant. However, since axis misalignment correction scales with the cosine of the angle between the footplate surface and reaction force, this effect must have been small. Indeed, even assuming shear force constituting 25% of the vertical component of reaction force (Kaufman et al. 1995), a decrease in axis misalignment correction and its contribution would be less than 10%. At time instants where axis misalignment correction was the dominant correction, a decrease in its contribution due to the non-orthogonality of reaction force would be marginal. Fourth, the ankle joint axis of rotation was assumed to pass through the transmalleolar midpoint parallel to the dynamometer axis of rotation. The actual ankle joint axis was shown to be oblique or skewed to the assumed axis and to change with ankle joint angle (Lundberg et al. 1989; Sheehan 2010). However, the instantaneous helical axis can be satisfactorily approximated by a fixed axis (Wade et al. 2019, 2020). Therefore, it can be assumed that a possible error due to a mismatch between the helical axis and the axis passing through the transmalleolar midpoint would be a systematic error.

## Conclusions

We demonstrated that errors in the assessment of active ankle joint moment during dynamic plantarflexions and the contributions of moment corrections have a high inter-individual variability. The inertial moment of the dynamometer adapter and axis misalignment were shown to be important contributors to differences between measured and active ankle joint moments. Inertial moment was shown to contribute more to measurement errors with increasing angular velocity and muscle activation level while axis misalignment was shown to contribute more with decreasing angular velocity and increasing muscle activation. Particularly, inertial moment and axis misalignment may represent a main source of error at higher and lower angular velocities, respectively, and tightly interact with each other at intermediate angular velocities and submaximal activation levels. Neglecting these corrections may lead to substantial errors in the assessment of active joint moment in dynamic plantarflexions.
